# Supplementary material for: Genome-Wide Identification and Characterization of WRKY Transcription Factors and Their Expression Profile in Loropetalum chinense var. rubrum
Source: Plants (Basel). 2023 May 27;12(11):2131. doi: 10.3390/plants12112131 (PMC10255886; doi:10.3390/plants12112131)
Supplement: Supplementary file 1 [file plants-12-02131-s001.zip › Table.S4.pdf]

Table.S4 Duplication, Ka, Ks, and Ka/Ks for *LcWRKY* gene pairs

Gene Name	Gene ID	Gene Name	Gene ID	Ka	Ks	Ka/Ks	Selection pressure
LcWRKY1	augustus79532.t1	*LcWRKY4*	augustus61312.t1	0.02498318	0.034715228	0.719660559	Purifying selection
LcWRKY11	augustus67305.t1	*LcWRKY13*	augustus31185.t1	0.002126907	0.016834374	0.126343086	Purifying selection
LcWRKY12	augustus31214.t1	*LcWRKY52*	augustus66669.t1	0.369018092	1.024307567	0.360261024	Purifying selection
LcWRKY13	augustus31185.t1	*LcWRKY50*	augustus44279.t1	0.256472607	1.63136428	0.157213573	Purifying selection
LcWRKY15	augustus75016.t1	*LcWRKY46*	augustus66513.t1	0.299605622	2.352623336	0.127349592	Purifying selection
LcWRKY20	augustus53007.t1	*LcWRKY53*	augustus17190.t1	0.181129467	2.048545976	0.088418551	Purifying selection
LcWRKY20	augustus53007.t1	*LcWRKY54*	augustus44319.t1	0.180381186	2.102938462	0.08577578	Purifying selection
LcWRKY21	augustus35146.t1	*LcWRKY35*	augustus63571.t1	0.427468958	2.261844085	0.188991346	Purifying selection
LcWRKY29	augustus78712.t1	*LcWRKY30*	augustus79284.t1	0.011443324	0.019523879	0.586119404	Purifying selection
LcWRKY36	augustus43450.t1	*LcWRKY43*	augustus64738.t1	0.286725314	1.478600123	0.193916739	Purifying selection
LcWRKY43	augustus64738.t1	*LcWRKY44*	augustus03162.t1	0.009337604	0.032563254	0.28675279	Purifying selection
LcWRKY48	augustus34353.t1	*LcWRKY49*	augustus63821.t1	0.001587722	0.010353918	0.153345068	Purifying selection
LcWRKY51	augustus55937.t1	*LcWRKY52*	augustus66669.t1	0.002300321	0	NaN	NaN
LcWRKY53	augustus17190.t1	*LcWRKY54*	augustus44319.t1	0.001201442	0.024392394	0.04925478	Purifying selection
LcWRKY57	augustus46502.t1	*LcWRKY25*	augustus70200.t1	0.284293687	0.798971022	0.355824779	Purifying selection
LcWRKY58	augustus68560.t1	*LcWRKY60*	augustus48988.t1	0	0.014888826	0	Purifying selection
LcWRKY6	augustus43926.t1	*LcWRKY7*	augustus18960.t1	0.001267963	0	NaN	NaN
LcWRKY6	augustus43926.t1	*LcWRKY55*	augustus13809.t1	0.152287012	0.912190329	0.166946533	Purifying selection
LcWRKY61	augustus49058.t1	*LcWRKY62*	augustus39209.t1	0.001177394	0.016713784	0.070444509	Purifying selection
LcWRKY61	augustus49058.t1	*LcWRKY74*	augustus24154.t1	0.286436779	1.430481649	0.200237996	Purifying selection
LcWRKY62	augustus39209.t1	*LcWRKY73*	augustus62150.t1	0.286578631	1.396253594	0.205248268	Purifying selection
LcWRKY63	augustus61539.t1	*LcWRKY13*	augustus31185.t1	0.360263976	1.57920481	0.228129989	Purifying selection
LcWRKY64	augustus39108.t1	*LcWRKY51*	augustus55937.t1	0.306391217	0.973586901	0.314703512	Purifying selection
LcWRKY65	augustus38604.t1	*LcWRKY12*	augustus31214.t1	0.348188556	0.928671281	0.374931973	Purifying selection
LcWRKY65	augustus38604.t1	*LcWRKY52*	augustus66669.t1	0.308817177	0.973586901	0.317195287	Purifying selection
LcWRKY67	augustus16677.t1	*LcWRKY78*	augustus37479.t1	0.253567279	0.938903425	0.270067477	Purifying selection
LcWRKY67	augustus16677.t1	*LcWRKY8*	augustus34013.t1	0.263201474	1.024989758	0.256784492	Purifying selection
LcWRKY68	augustus16954.t1	*LcWRKY69*	augustus69059.t1	0.034587199	0.05515315	0.627111933	Purifying selection
LcWRKY68	augustus16954.t1	*LcWRKY77*	augustus38956.t1	0.257328614	0.952431781	0.270180626	Purifying selection
LcWRKY7	augustus18960.t1	*LcWRKY55*	augustus13809.t1	0.153953378	0.908176474	0.169519232	Purifying selection
LcWRKY73	augustus62150.t1	*LcWRKY74*	augustus24154.t1	0.00119024	0.008409338	0.141537924	Purifying selection
LcWRKY78	augustus37479.t1	*LcWRKY8*	augustus34013.t1	0.315027757	1.180309387	0.266902696	Purifying selection
