# Supplementary figures and images for: Genome-Wide Identification and Characterization of WRKY Transcription Factors and Their Expression Profile in Loropetalum chinense var. rubrum
Source: Plants (Basel). 2023 May 27;12(11):2131. doi: 10.3390/plants12112131 (PMC10255886; doi:10.3390/plants12112131)

Motif 1

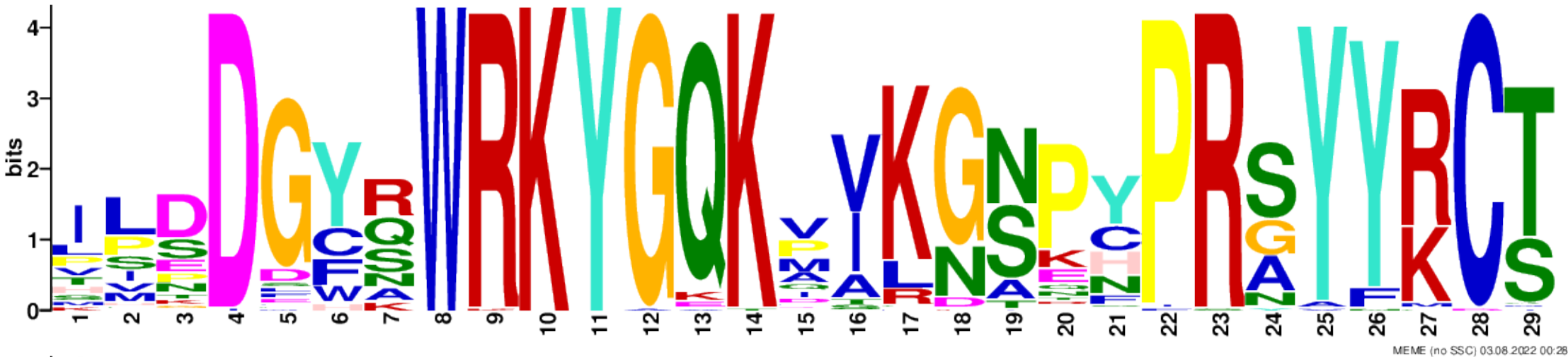

Motif 2

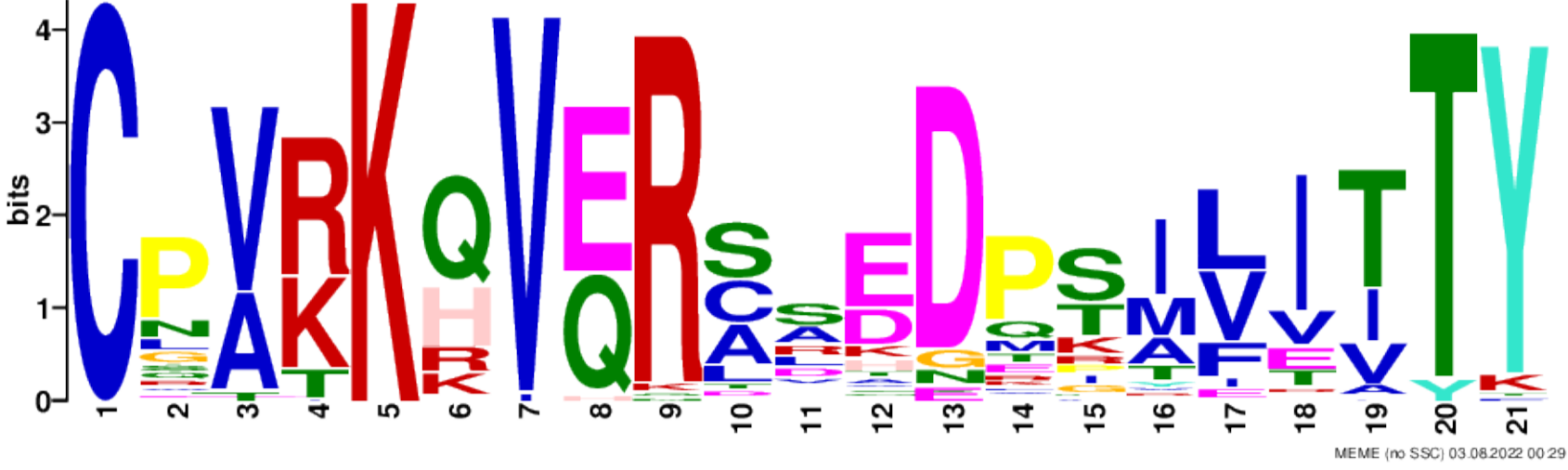

Motif 3

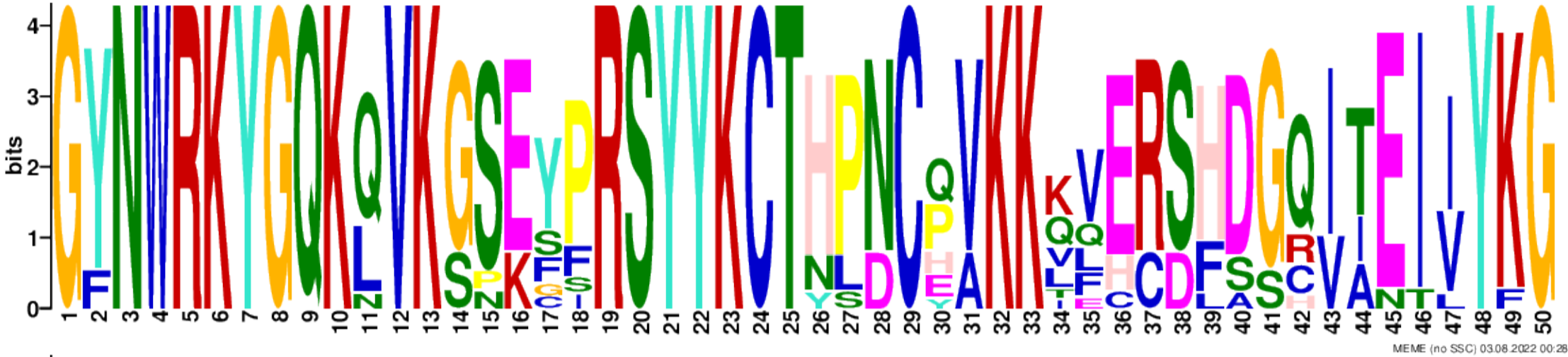

Motif 4

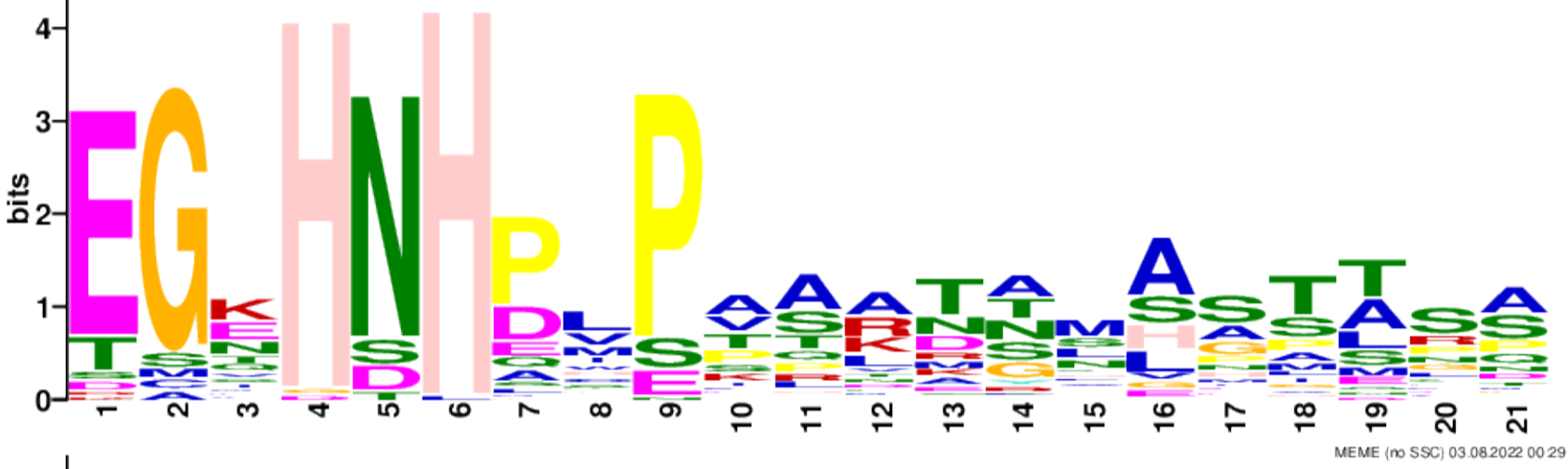

Motif 5

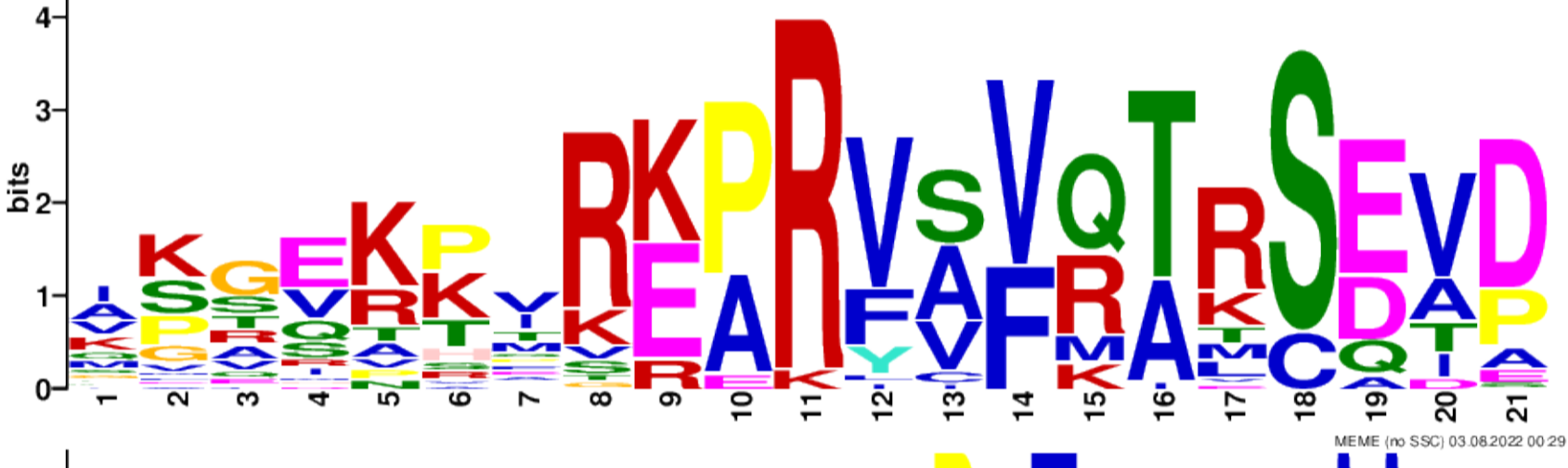

Motif 6

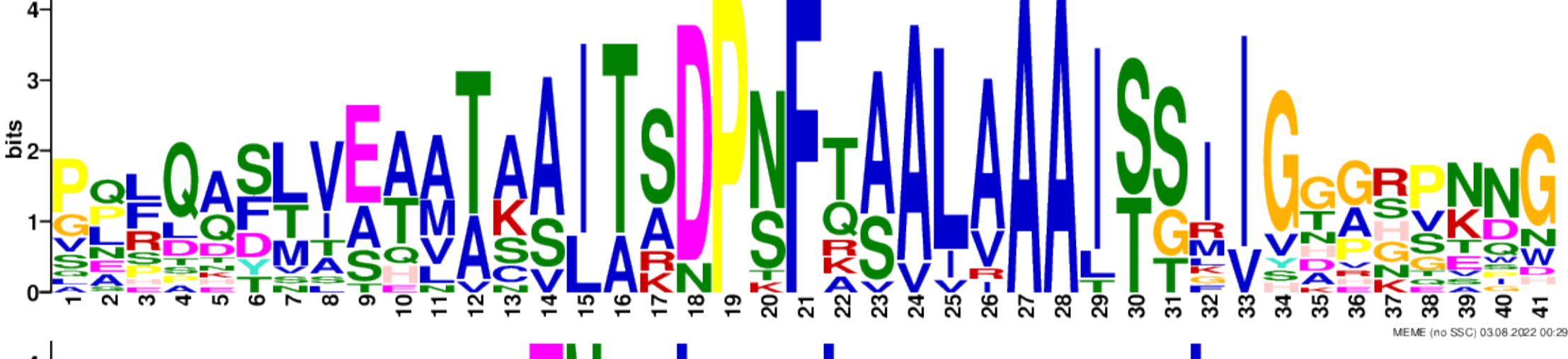

Motif 7

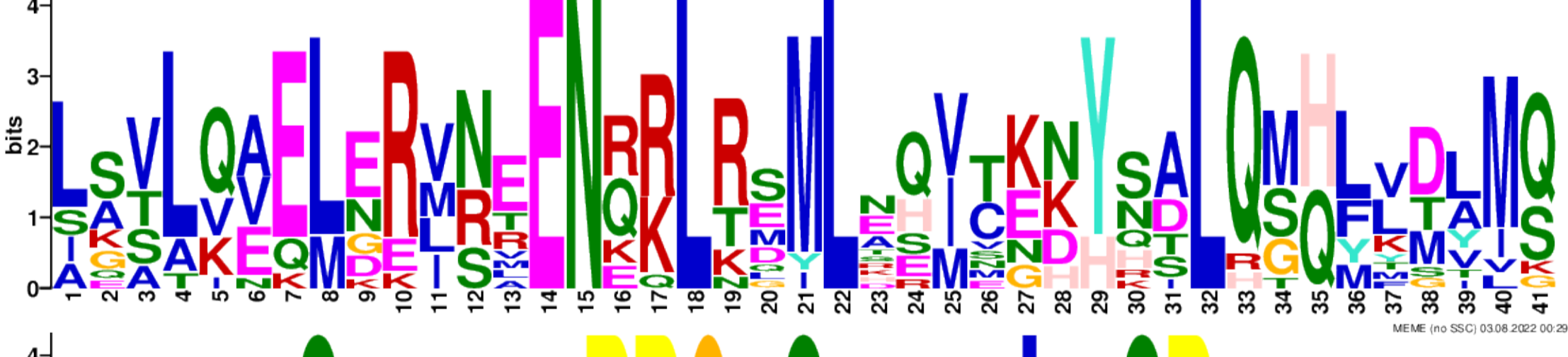

Motif 8

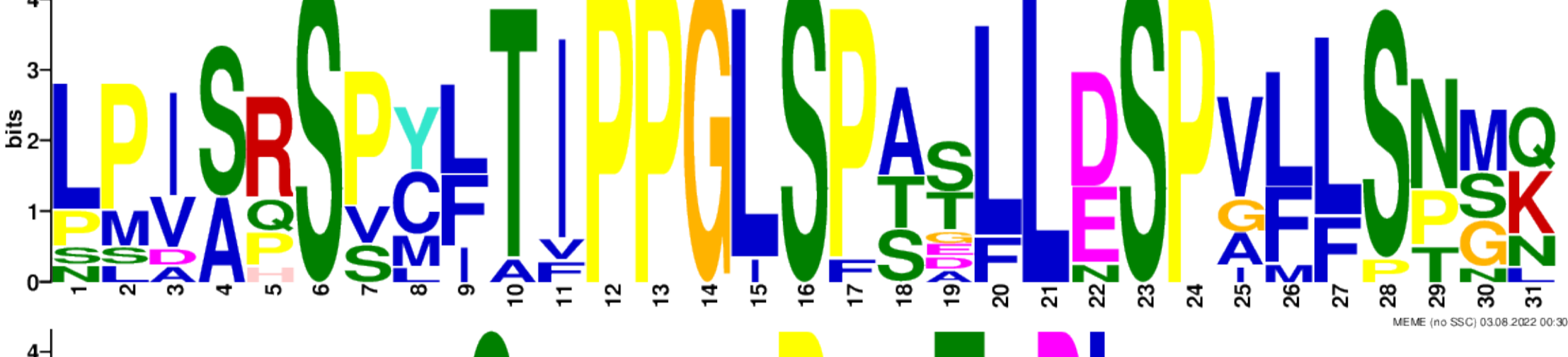

Motif 9

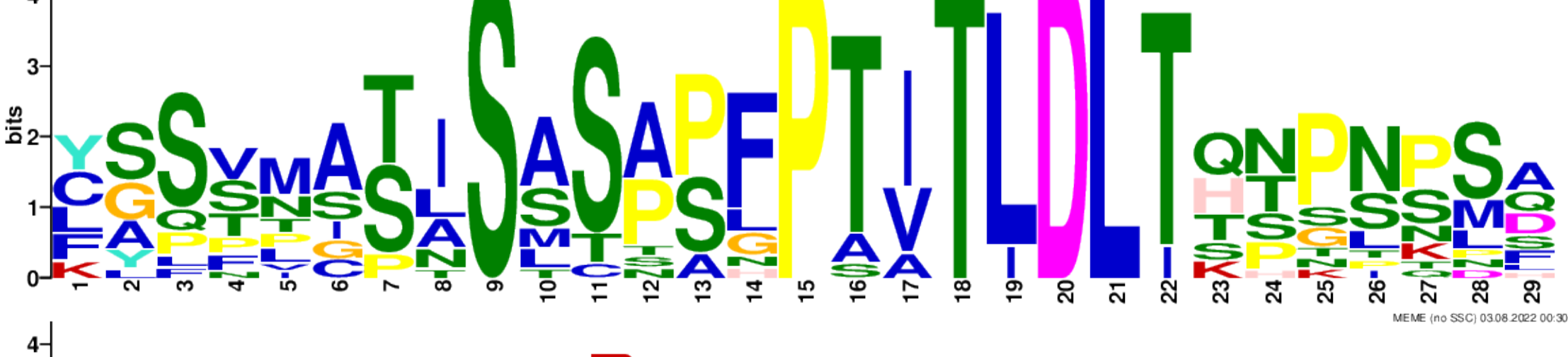

Motif 10

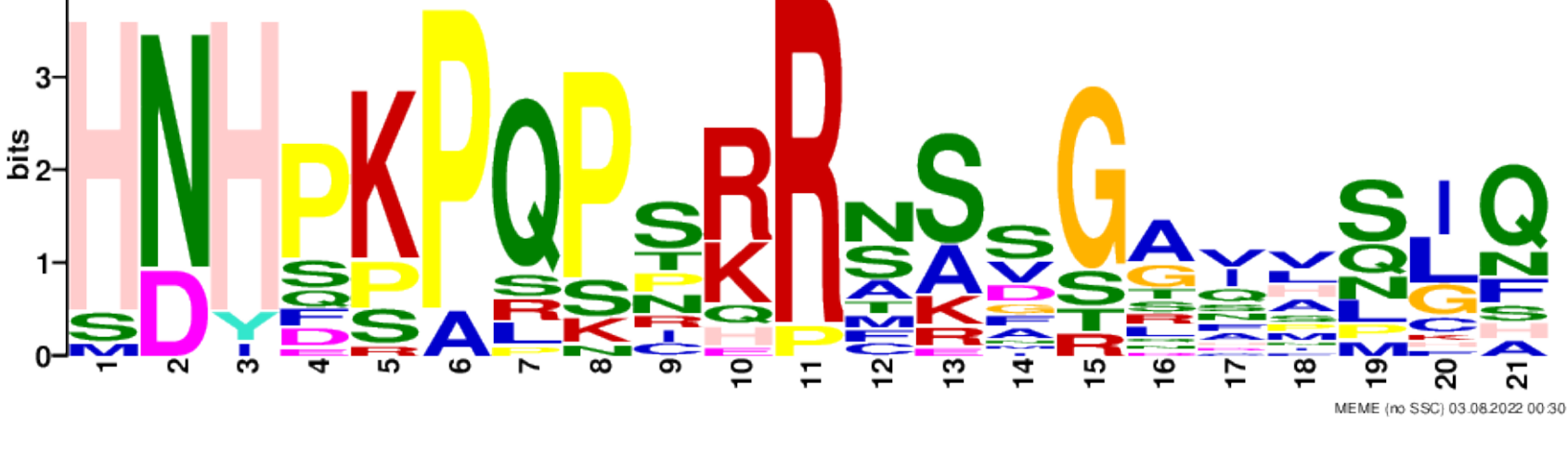

Figure S1: motif analysis results

Supplement: Supplementary file 1 [file plants-12-02131-s001.zip › Figure S1.pdf]
